# A Graphene-Based Biosensing Platform Based on Regulated Release of an Aptameric DNA Biosensor

**DOI:** 10.3390/s151128244

**Published:** 2015-11-09

**Authors:** Yu Mao, Yongli Chen, Song Li, Shuo Lin, Yuyang Jiang

**Affiliations:** 1 Laboratory of Chemical Genomics, School of Chemical Biology and Biotechnology, Peking University Shenzhen Graduate School, Shenzhen 518055, China; E-Mails: maoyu_gt@126.com, (Y.M.); chenyongli0617@163.com (Y.C.); lisong@pkusz.edu.cn (S.L.); shuolin@ucla.edu (S.L.); 2 The Ministry-Province Jointly Constructed Base for State Key Lab-Shenzhen Key Laboratory of Chemical Biology, the Graduate School at Shenzhen, Tsinghua University, Shenzhen 518055, China; 3 Department of Molecular, Cell and Developmental Biology, University of California, Los Angeles, CA 90095, USA

**Keywords:** aptamer, DNA biosensor, graphene oxide, Isothermal amplification

## Abstract

A novel biosensing platform was developed by integrating an aptamer-based DNA biosensor with graphene oxide (GO) for rapid and facile detection of adenosine triphosphate (ATP, as a model target). The DNA biosensor, which is locked by GO, is designed to contain two sensing modules that include recognition site for ATP and self-replication track that yields the nicking domain for Nt.BbvCI. By taking advantage of the different binding affinity of single-stranded DNA, double-stranded DNA and aptamer-target complex toward GO, the DNA biosensor could be efficiently released from GO in the presence of target with the help of a complementary DNA strand (CPDNA) that partially hybridizes to the DNA biosensor. Then, the polymerization/nicking enzyme synergetic isothermal amplification could be triggered, leading to the synthesis of massive DNA amplicons, thus achieving an enhanced sensitivity with a wide linear dynamic response range of four orders of magnitude and good selectivity. This biosensing strategy expands the applications of GO-DNA nanobiointerfaces in biological sensing, showing great potential in fundamental research and biomedical diagnosis.

## 1. Introduction

Due to the obvious advantages, such as highly specific Watson–Crick base pairing interactions, and single-stranded flexibility, DNA is widely employed as a smart building block in the design of DNA-based biosensors [1,2,3,4,5]. The efficient construction of DNA biosensors has become a more and more active research area in biosensing applications. Recently, aptamer-based DNA biosensor by multiple processes of replication, nicking, and strand displacement has attracted considerable attention for its excellent isothermal amplification capability and relative high sensitivity and specificity [6,7,8].

However, only the structure-switched aptamers have been employed in that system.

Aptamers are single-stranded DNA or RNA sequences that can be isolated from random-sequence libraries using *in vitro* selection techniques [9,10]. They can bind to their targets with high affinity and specificity. Since the discovery of the first aptamer in 1990, various aptamers have been evaluated *in* *vitro* against many classes of molecules such as proteins [11], small molecules [12], and even cells [13]. Therefore, aptamer-based biosensors have been widely studied due to their high sensitivity and selectivity to almost all types of ligands. It has been well documented that during the recognition of the target molecules, aptamers and their cognate targets can form different well-defined structures [14,15] that could be applied for biosensor design and analytical applications. However, some aptamers have their secondary structure preformed in the unbound state with only tertiary structure change occurring during target binding or there is only a small amount of energy change between the free and complexed state of aptamers which result in a relatively high background. Some effects have been made to eliminate the undesired signal production in the absence of the target, such as using a DNA sequence as a blocker [6,8,16] or splitting the aptamer into two parts [17,18]. However, those strategies make the design of oligonucleotide sequences more complex.

Graphene oxide (GO), a two-dimensional nanomaterial, has been extensively studied due to its high physicochemical activity, large surface to volume ratio, water dispersibility, as well as good biocompatibility [19,20,21,22,23,24]. In particular, it has been found that GO can bind single-stranded DNA (ssDNA) nonspecifically via hydrophobic and π-π stacking interactions between the nucleobases and GO [25]. Thus, many DNA-GO based biosensors have been designed using GO as a blocker for the detection of targets that include DNA [26,27], microRNA [28], metal ions [29], small molecules [30], and proteins [31]. Several groups have developed amplification strategies to enhance the sensitivity of DNA-GO based sensors [32,33]. However, DNA biosensors, which contain long ssDNA sequences, may not be generally applicable to the GO based sensing platform, as the binding interaction of ssDNA to GO typically becomes greater with the increasing sequence length [34]. For that reason, the relatively stronger GO adsorption from longer ssDNA is likely to out-compete the formation of aptamer-target complex, especially when dealing with low-affinity aptamers. Recently, Li group presented a generalizable sensing strategy based on regulated GO adsorption that can accommodate RNA aptamers of various lengths [35]. The remarkable achievement made in the development of GO adsorption method toward longer RNA aptamers validates the potential compatibility of long length DNA biosensors with graphene materials.

**Figure 1:**
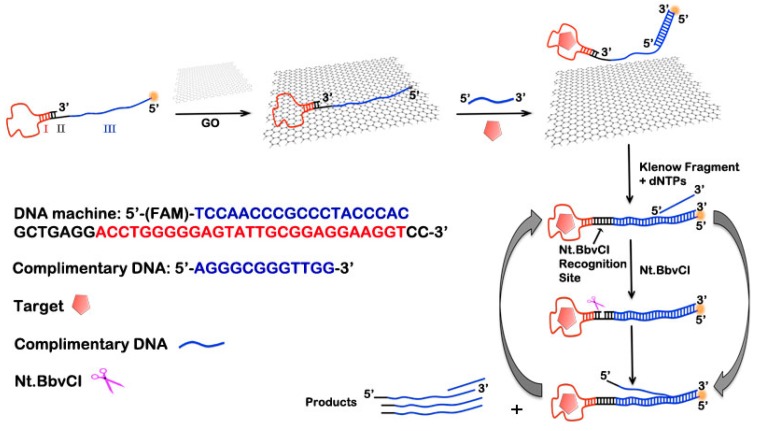
Schematic illustration of the graphene-based biosensing platform based on regulated release of an aptameric DNA biosensor and the amplification detection of target (ATP). This involves two principal steps: (1) the effective absorption of DNA biosensor by GO and regulated release of the DNA biosensor from GO in the presence of target; and (2) the polymerization/nicking enzyme synergetic isothermal amplification whose products can be detected by adding in SYBR Gold.

In the present work, by taking advantage of the preferential binding to ssDNA over dsDNA and aptamer-target complex of GO, an aptameric DNA biosensor coupled with GO-based sensing platform is developed using ATP as the trigger. The integration of a DNA recognition element and signal amplification probe into a bifunctional probe is proposed. In our design, the FAM (Carboxyfluorescein)-labeled hairpin aptameric DNA biosensor is first adsorbed onto GO, which results in fluorescence quenching due to the close proximity of the fluorophore to GO [36]. The fluorescent labeling here is used to measure whether the DNA biosensor is completely absorbed by GO. Then a complementary DNA strand (CPDNA) is hybridized to the 5′-end of the DNA biosensor, which weakens DNA biosensor-GO interaction. Upon the addition of target, the sensing system becomes completely separated from GO. Then the replication cycles of polymerization, nicking, and strand displacement has been triggered, leading to the synthesis of a large amount of sequence units that can be easily detected (Figure 1). The programmability of nucleic acid sequences and catalytic efficiency of biomolecules (polymerases, endonucleases, and nicking enzymes) coupled with the regulated DNA biosensor-GO adsorption strategy may enhance the generality of these methods and pave the way for the application of GO-based DNA biosensing.

## 2. Experimental Section

### 2.1. Materials and Reagents

Klenow fragment polymerase exo^-^ (KF polymerase, 5 U/μL), nicking endonuclease Nt.BbvCI (10 U/μL) and 10 × NEBuffer2 (500 mM NaCl, 100 mM Tris-HCl, 100 mM MgCl_2_, 10 mM DTT, PH 7.9) were purchased from New England Biolabs (NEB, Beijing, China). Adenine (A), thymine (T), guanine (G), cytosine (C) and deoxynucleotide triphosphates (dNTPs, 2.5 mM) were obtained from Takara Biotechnology Co. Ltd. (Dalian, China). SYBR Gold (10,000 × concentrated stock in DMSO) was purchased from Sigma-Aldrich (St. Louis, MO, USA). Graphite oxide was obtained from Nanjing XFNANO Materials Tech Co. Ltd. (Nanjing, China). Human serum samples were obtained from Peking University Shenzhen Hospital. 10 × TBE was made in house with the following recipe (for 1 L): 108 g Tris base, 55 g boric acid, 20 mL of 0.5 M EDTA (pH 8.0). Water was purified with a Milli-Q Synthesis from a Millipore system. All DNA oligonucleotides were synthesized by Invitrogen Bio Inc. (Shanghai, China) and their sequences are shown in Table S1. All DNA oligonucleotides were purified by 10% denaturing PAGE before use except that the FAM-labeled DNA was purified by HPLC. All other chemicals involved were of analytical reagent grade and used without further purification.

### 2.2. Apparatus

All fluorescence anisotropy measurements were carried out in a 96-well assay plate (Costar, Washington, DC, USA) using a microplate reader (Tecan infinite M1000 Pro, Männedorf, Switzerland). The slits for excitation and emission were both set at 5.0 nm and the excitation voltage was 700 V. The excitation wavelength was set at 485 nm, and the emission spectra from 500 to 600 nm were collected. The fluorescence intensity at 520 nm (for FAM) and 544 nm (for SYBR Gold) was used to evaluate the performance of the proposed strategy. All fluorescence detections were carried out at room temperature (RT).

### 2.3. Adsorption and Regulated Release of the DNA Biosensor from Graphene Oxide Surface

Thirty microliters of 10 × NEBuffer2, 180 μL of H_2_O, 30 μL of 1 μM FAM-labeled DNA biosensor, and 60 μL of 50 μg/mL GO solution were incubated at RT for 30 min. The final DNA biosensor concentration was 100 nM, whereas the final GO concentration was 10 μg/mL. Under this condition, the DNA biosensor is completely adsorbed by GO (Figure S1). Then, the following was introduced: (i) CPDNA only; (ii) target only; or (iii) CPDNA and target. The reaction mixture was incubated at RT for 1 h, and then centrifuged for 10 min at 12,000 g to remove the GO. The supernatant (100 μL) was taken out for analysis. Note that the reaction tube was wrapped with aluminum foil to prevent photo-bleaching. The fluorescence readings were measured at RT in a 96-well assay plate (Costar, Washington, DC, USA) using a microplate reader (Tecan infinite M1000 Pro, Switzerland) with λex/λem of 485/520 nm.

### 2.4. Detection of ATP by Using the Biosensing Platform

In a typical ATP detection experiment, 10 μL of 10 × NEBuffer2, 60 μL of H_2_O, 10 μL of 1 μM FAM-labeled DNA biosensor, and 20 μL of 50 μg/mL GO solution were incubated at room temperature (RT) for 30 min. Then, 1 μL of CPDNA (10 μM, final concentration: 100 nM) and 5 μL of ATP stock solution (0–100 mM) were added, following 1 h incubation at RT. Subsequently, the solution was centrifuged for 10 min at 12,000 g to remove the GO. The supernatant (10 μL) was transferred into a 1.5 mL microcentrifuge tube. Next, 1 μL of 10 × NEBuffer2, 2 U of Klenow fragment, 8 U of Nt.BbvCI, and 4 μL of dNTPs (with a final concentration of 500 μM) were introduced to the above mixture (total volume: 20 μL). The reaction mixture was incubated at 37 °C for 1 h (Figure S2) before heating at 90 °C for 10 min. Finally, 10 μL of the products from the above reaction mixture was added into 10 μL of 10 × SYBR Gold, 10 μL of 10 × TBE and 70 μL H_2_O. Note that the reaction tube was wrapped with aluminum foil to prevent photo-bleaching. The mixture was incubated at RT for 5 min and the fluorescence readings were measured at RT in a 96-well assay plate (Costar, Washington, DC, USA) using a microplate reader (Tecan infinite M1000 Pro, Switzerland) with λex/λem of 485/544 nm.

For measurements in 10% human serum, different ATP concentrations were spiked in the GO-based aptasensor solution containing 10% human serum instead of buffer alone, and the other processes were same as in buffer detection.

### 2.5. Selectivity Investigation of the Proposed Biosensing Platform

The experimental procedure for selectivity test is identical to that used for ATP detection except that guanosine triphosphate (GTP), cytidine triphosphate (CTP), uridine triphosphate (UTP) or the mixture of them was added in to the biosensing platform respectively instead of ATP.

## 3. Results and Discussion

### 3.1. Design of the GO-Based DNA Biosensor and Polymerization/Nicking Enzyme Synergetic Isothermal Amplification Principle

The design of the DNA biosensor and the operation mechanism of the amplification system are illustrated in Figure 1, in which ATP is adopted as the model target. In the aptameric DNA biosensor, the hairpin target recognition probe and track DNA are integrated into one DNA molecule. The DNA biosensor consists of three regions: ATP-binding aptamer (Region I), Nt.BbvCI recognition site (Region II) and the amplification track (Region III). The undesired activation of the DNA biosensor is blocked by GO surface via π-π stacking interactions between GO and ssDNA. This strategy of GO through noncovalent adsorption of the ssDNA possesses the simplicity of functionalization without the need of any coupling reagents. A complementary DNA strand (CPDNA) is then introduced. CPDNA is designed to partially hybridize to the 5′-end of the DNA biosensor. The addition of a complementary sequence weakens DNA biosensor-GO interaction, and enables the sensing system completely separated from GO upon the addition of target. Finally, the enzyme network makes the replication of track from the 3′-end of the DNA biosensor over the region II and region III occur. The spontaneous cycle of replication, scission and displacement leads to the formation of massive DNA amplicons that are complimentary to region III. Consequently, the aptamer-target binding event is efficiently amplified. Finally, the fluorescent DNA dye SYBR Gold is added into the reaction system, resulting in a strong fluorescence signal increase that can be easily detected.

### 3.2. Feasibility of the Biosensing Platform for ATP Detection

**Figure 2:**
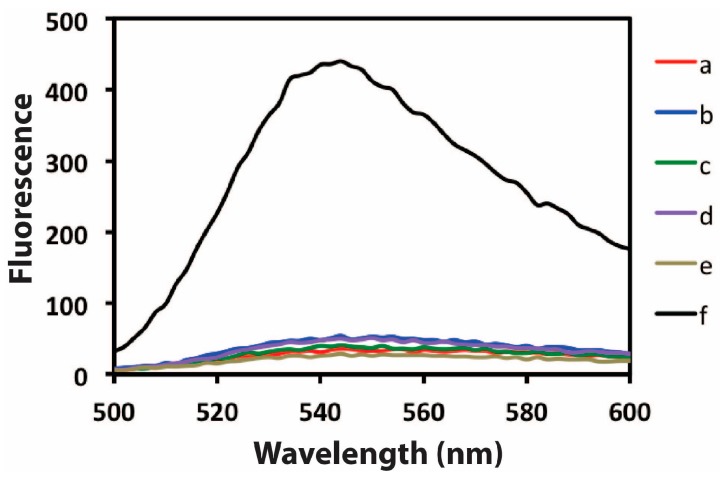
The validity of GO-based DNA biosensor for the amplification detection of target ATP: fluorescence spectrum of (**a**) GO-based DNA biosensor system consisting of target, polymerase and nicking enzyme in the absence of CPDNA; (**b**) GO-based DNA biosensor system consisting of CPDNA, polymerase and nicking enzyme in the absence of target; (**c**) GO-based DNA biosensor system consisting of target, CPDNA, nicking enzyme in the absence of polymerase; (**d**) same as (**c**), but it contains polymerase instead of nicking enzyme; (**e**) only contain GO-based DNA biosensor system; and (**f**) same as (**b**), but in the presence of target. The concentration of target ATP involved is 500 μM.

The design of the biosensing platform and its operation principle are illustrated in Figure 1. The success of target-induced amplification and efficient suppression of unwanted polymerization in the absence of target are critical issues for the operation of the system. We separate the target recognition step from the amplification step; that is, because it is known that the ATP aptamer can also bind dATP. To evaluate the feasibility of the proposed aptameric DNA biosensor, the behavior of the biosensing platform is investigated under different conditions. As shown in Figure 2, an extremely low fluorescence emission can be observed at 544 nm for the single signaling aptameric DNA biosensor (Line e). This demonstrates the high GO adsorption efficiency towards the DNA biosensor. The addition of target ATP, CPDNA, polymerase and nicking enzyme can cause a significant change in the fluorescence intensity at 544 nm (Line f). In the absence of the target ATP (Line b), there is no increase of the fluorescence peak. Similarly, when the CPDNA is not involved in the biosensor system (Line a), there is little difference of fluorescence peak between it and the blank. Line a and Line b indicate that the binding interaction of the aptameric DNA biosensor to GO out-competes the formation of aptamer-target complex. The addition of CPDNA weakens DNA biosensor-GO interaction, while still keeping the DNA biosensor adsorbed on the GO surface. There is no amplified signal in the absence of KF polymerase (Line c) or in the absence of Nt.BbvCI nicking enzyme (Line d), demonstrating that both of the enzyme is crucial to the autonomous amplification of the DNA biosensor. The measured data offer unmistakable evidence that the aptameric DNA biosensor coupled with polymerase/nicking enzyme synergetic isothermal amplification capability can be employed in GO-based sensing platform.

### 3.3. Absorption and Regulated Release of the DNA Biosensor

Whether the DNA biosensor could be effectively adsorbed by GO is crucial for the design of the GO-based biosensing platform, which was firstly optimized in this work. The fluorescence changes of the FAM-labeled DNA biosensor were recorded after exposure to various amounts of graphene oxide in order to determine the concentration of graphene oxide required to adsorb 100 nM FAM-labeled DNA biosensor. The maximal fluorescence quenching was observed when graphene oxide reached 10 μg/mL (Figure S1). Therefore, 10 μg/mL GO was used for the rest of the experiments.

**Figure 3:**
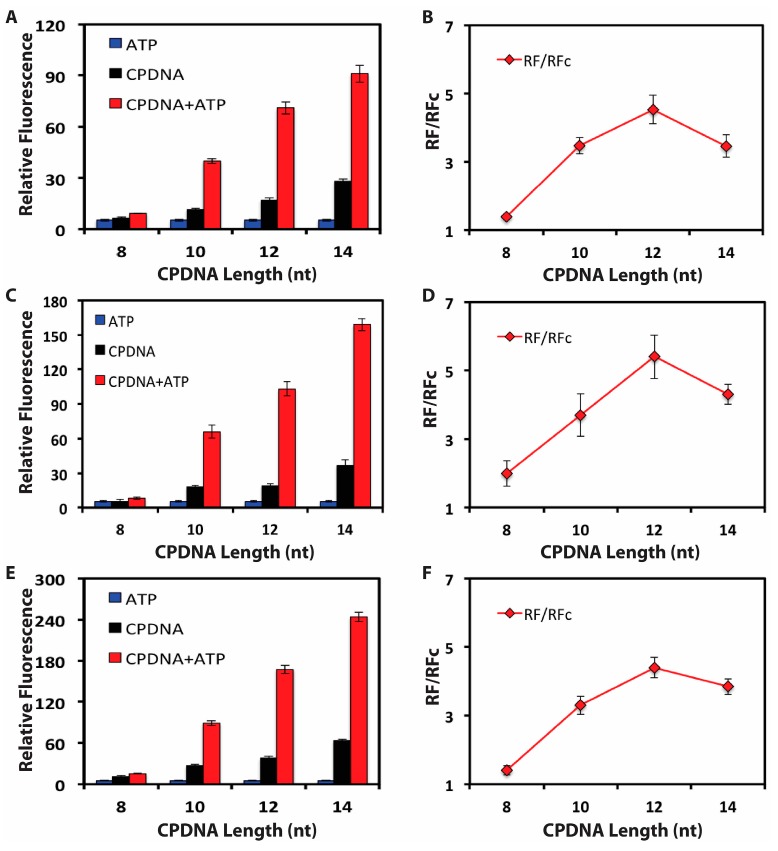
Optimization of the complementary DNA (CPDNA) length (nucleotides, nt) and concentration. FAM-labeled DNA biosensor-GO was incubated for 1 h, then target ATP (blue, 500 μM), CPDNA (black), or a combination of CPDNA + target (red) was added. Relative Fluorescence (RF) was plotted after 1 h. RF = F_T_ − F_B_, where F_T_ is the fluorescence reading of a test mixture; F_B_ is the fluorescence reading of the blank mixture. (**A**) CPDNA: 10 nM; (**B**) RF/RFc *vs.* CPDNA length, where RF is the red plot in (**A**) and RFc is the blank plot in (**A**); (**C**) CPDNA: 100 nM; (**D**) RF/RFc *vs.* CPDNA length, where RF is the red plot in (**C**) and RFc is the blank plot in (**C**); (**E**) CPDNA: 1000 nM; and (**F**) RF/RFc *vs.* CPDNA length, where RF is the red plot in (**E**) and RFc is the blank plot in (**E**). The data are an average of three independent experiments.

The single-stranded DNA biosensor is adsorbed onto GO surface via a strong π-π stacking interaction. CPDNA hybridizes to the 5′-end of the DNA biosensor making this part of DNA biosensor cannot be adsorbed on GO surface due to the efficient shielding of nucleobases within the negatively charged phosphate backbone of double-stranded DNA, thus weakens the interaction between GO and the DNA biosensor. So the length and concentration of the CPDNA play an important role in regulated releasing of the DNA biosensor from GO upon the addition of target. We designed various lengths of CPDNA (8–14 nt) and tested various CPDNA concentrations to optimize the releasing efficiency of the DNA biosensor (Figure 3). For all CPDNA lengths, we found that the addition of target and CPDNA together significantly enhanced fluorescent signal, as compared to the addition of CPDNA or target only. The longer length of the CPDNA increased the releasing of DNA biosensor together with the target. However, when CPDNA reached 14 nucleobases, it could release the DNA biosensor without the target, thus increased the background. Particularly, 100 nM CPDNA which has a length of 12 nt (CPDNA12) generated the highest signal to noise ratio (S/N) and was chosen for further analysis.

### 3.4. Quantitative Detection of ATP

**Figure 4:**
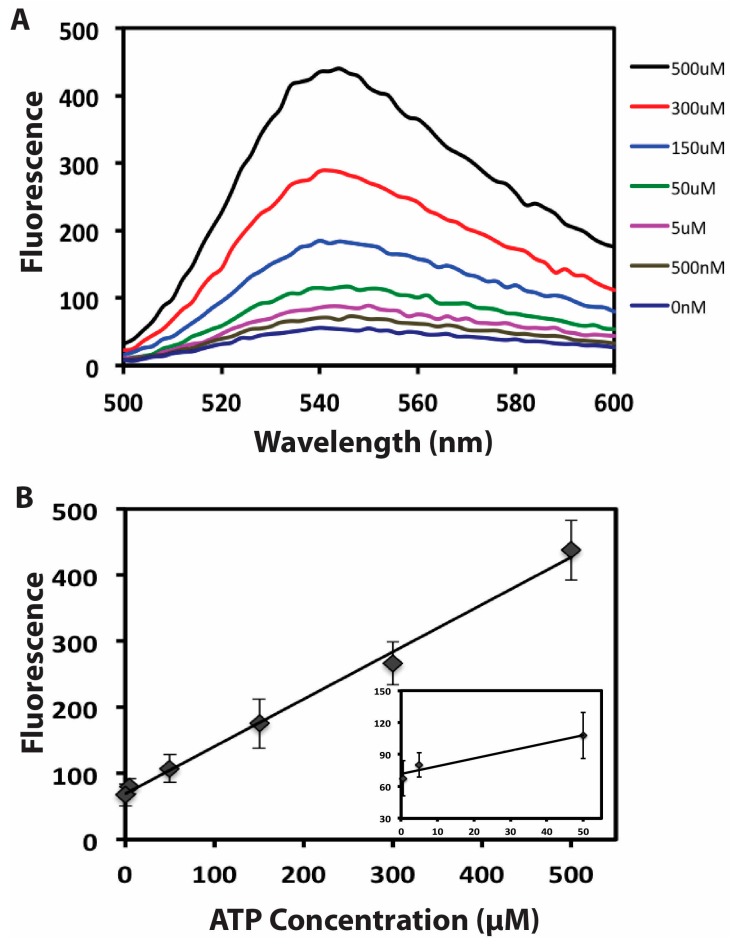
(**A**) Fluorescence-emission spectrum for the amplification aptameric DNA biosensor at a 1 h interval in the presence of different concentrations of target ATP; (**B**) Calibration curve in the standard ATP concentration range of 0.5–500 μM. The inset shows the linearity of the fluorescence response for the sensing system against the low target concentrations. Each data point represents the average value of three independent experiments.

In order to confirm the capability of the proposed GO-based aptameric DNA biosensor to quantify target molecular, different concentrations of ATP were analyzed. Under the optimized experimental conditions, it can be seen that the fluorescence intensity of the system increased with the increased concentration of ATP (Figure 4A). The increased fluorescence emission could be ascribed to the releasing of DNA biosensor induced by ATP binding, thus producing more amplified products. The plot of the fluorescence intensity change of the system in response to various concentrations of ATP is exhibited in Figure 4B. Notably, good linearity can be observed in a broad concentration range (from 0.5 to 500 μΜ ATP, R = 0.995) of ATP. The detection limit of ATP was estimated to be 380 nM based on the criterion of three times of the standard deviation of the blank. Such detection limit is comparable or better than most of the other GO-based or amplified ATP assay methods [37,38,39,40,41,42]. The results demonstrate that highly sensitive detection of ATP can be realized by the new biosensing strategy. Besides the high sensitivity, the broad concentration range is sufficient to cover the physiological concentration range for ATP in human plasma [43]. These results suggest that the present method could potentially be used for ATP detection in human plasma.

### 3.5. Selective Measurement of ATP

**Figure 5:**
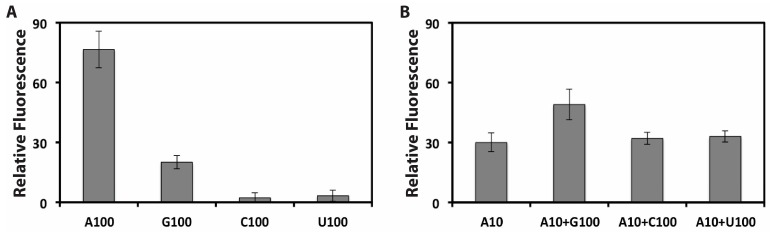
(**A**) Relative Fluorescence (RF) changes in the presence of ATP (100 μM), GTP (100 μM), CTP (100 μM), and UTP (100 μM), respectively; (**B**) RF changes in the presence of ATP (10 μM), ATP (10 μM) + GTP (100 μM), ATP (10 μM) + CTP (100 μM), and ATP (10 μM) + UTP (100 μM), respectively. RF = F_T_ − F_B_, where F_T_ is the fluorescence reading of a test mixture and F_B_ is the fluorescence reading of the blank mixture. Each data point represents the average value of three independent experiments.

To evaluate the specificity of the proposed biosensing platform for target molecular, the aptasensor was challenged with other organic triphosphate analogues, such as GTP, CTP and UTP. Figure 5A shows the comparison between the relative fluorescence response intensities of ATP and other organic triphosphate analogues. As can be seen, the relative fluorescence intensities of present aptasensor for non-specific binding from GTP (100 μM), CTP (100 μM), and UTP (100 μM) are much smaller than that caused by the specific binding of ATP (100 μM). To further study the specificity of the system, ATP detection was investigated by testing ATP (10 μM) in the presence of other small molecules (each 100 μM) including GTP, CTP and UTP. As can be seen in Figure 5B, 10 times higher concentrations of CTP or UTP only brought in less than 11% interference on the detection of ATP. While 10 times higher concentrations of GTP brought in more than 50% interference on the detection of ATP, this may be attributed to the similar structure between ATP and GTP. These results clearly indicated that this biosensing platform provides an acceptable selectivity for target molecule.

### 3.6. Application of the Biosensing Platform in Real Sample

To evaluate its feasibility in practical samples, this method was applied to ATP detection in concentrated (10%) human serum. Four different ATP concentrations were added into the GO-based aptameric DNA biosensor solution containing 10% human serum instead of buffer alone, and other experimental conditions were same. The results (Figure S3) show that the sensor can still work in this complex matrix but has a shorter concentration range, suggesting that this method could potentially be utilized for analyzing biological samples. The sensitivity of the biosensing platform in real samples still needs to be improved and caution needs to be exercised for background signal, which will be studied in our future work.

## 4. Conclusions

In summary, the successful construction and characterization of GO-based aptameric DNA biosensor have been accomplished in this work. The authentic operation behavior is based on different binding affinity of the single-stranded aptameric DNA biosensor, double-stranded CPDNA and the aptamer-target complex toward GO. The aptameric DNA biosensor is locked by GO nonspecifically, which makes it easier to design the target recognition motif using aptamers that have a similar secondary structure preformed between the free and bound state. This design converts target detection to the production of massive DNA amplicons, giving rise to an enhanced sensitivity. The sensitivity could be further improved by introducing exponential amplification strategy. This can, therefore, overcome the problem of relatively poor detection sensitivity that is typical of low-affinity aptamers. Significantly, this method expands the applications of GO-DNA nanobiointerfaces in biosensing. Additionally, this paves the way to construct a variety of GO-based DNA sensing devices for diagnostics, biosensing and related applications.

## Supplementary Files

Supplementary File 1
