# Supplementary material for: A Graphene-Based Biosensing Platform Based on Regulated Release of an Aptameric DNA Biosensor
Source: Sensors (Basel). 2015 Nov 9;15(11):28244–56. doi: 10.3390/s151128244 (PMC4701278; doi:10.3390/s151128244)
Supplement: Supplementary File 1 [file sensors-15-28244-s001.pdf]

OPEN ACCESS

sensors

ISSN 1424-8220
www.mdpi.com/journal/sensors

Supplementary Information

A Graphene-Based Biosensing Platform Based on Regulated Release of an Aptameric DNA Biosensor. *Sensors* 2015, *15*, 28244-28256

Yu Mao [1,2], Yongli Chen [1,2], Song Li [1], Shuo Lin [1,3] and Yuyang Jiang [2,*]

[1] Laboratory of Chemical Genomics, School of Chemical Biology and Biotechnology, Peking University Shenzhen Graduate School, Shenzhen 518055, China; E-Mails: maoyu_gt@126.com, (Y.M.); chenyongli0617@163.com (Y.C.); lisong@pkusz.edu.cn (S.L.); shuolin@ucla.edu (S.L.)

[2] The Ministry-Province Jointly Constructed Base for State Key Lab-Shenzhen Key Laboratory of Chemical Biology, the Graduate School at Shenzhen, Tsinghua University, Shenzhen 518055, China

[3] Department of Molecular, Cell and Developmental Biology, University of California, Los Angeles, CA 90095, USA

* Author to whom correspondence should be addressed; E-Mail: jiangyy@sz.tsinghua.edu.cn; Tel./Fax: +86-755-2603-2094.

Table S1. DNA oligonucleotides used in the study.

Name	Sequence (5′-3′)
FAM-labeled DNA	(FAM)-TCCAACCCGCCCTACCCAC***GCTGAGG*** *ACCTGGGGGAGTATTGCGGAGGAAGGT*CC
CPDNA8	CGGGTTGG
CPDNA10	GGCGGGTTGG
CPDNA12	AGGGCGGGTTGG
CPDNA14	GTAGGGCGGGTTGG

The segments shown in bold-italic letters are the Nt.BbvCI recognition sequence and underlined-italic letters are the ATP aptamer sequence.

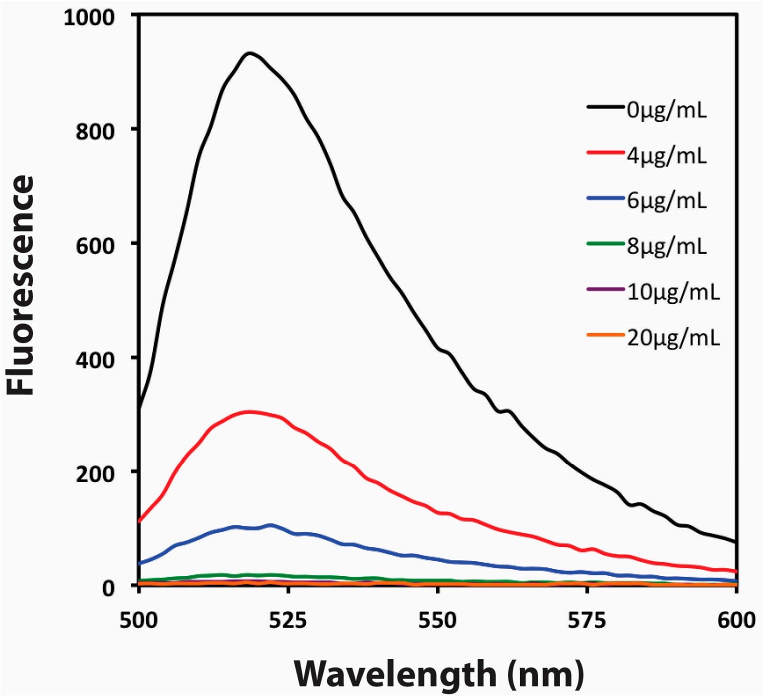

Figure S1. GO concentration testing for fluorescence quenching. FAM-labeled DNA biosensor (100 nM) was treated with different GO concentrations (0, 4, 6, 8, 10, 20 μg/mL). Fluorescence spectra were measured after 1 h incubation.

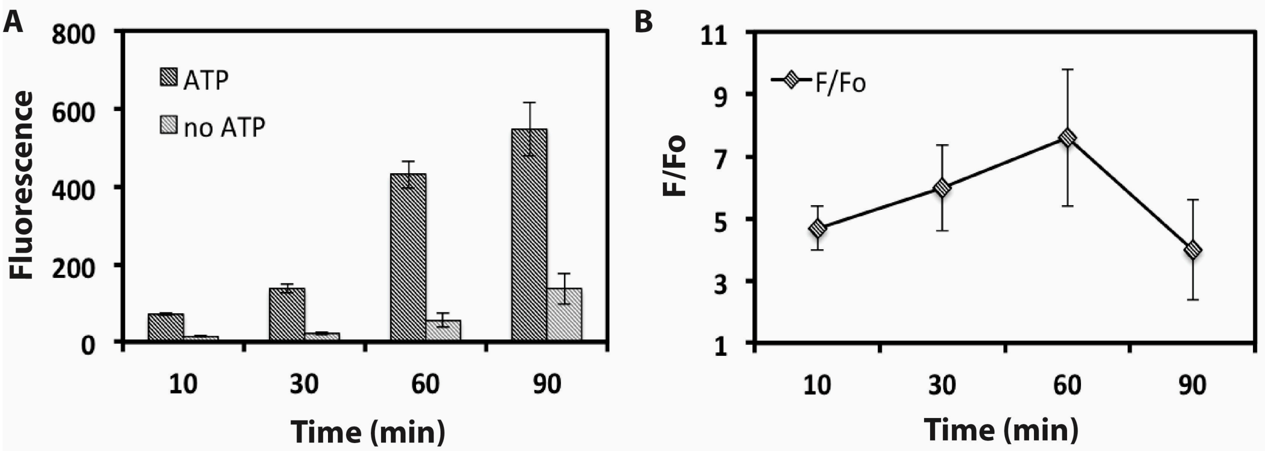

Figure S2. Time-dependent fluorescence responses. (**A**) The fluorescence *vs.* polymerization/nicking enzyme synergetic isothermal amplification time in the presence and absence of ATP; (**B**) F/F_0 *vs.* the polymerization/nicking enzyme synergetic isothermal amplification time, where F is the fluorescence reading in the presence of ATP and F_0 is the fluorescence reading in the absence of ATP. The ATP used in (**A**,**B**) is 500 μM. The error bar represents the standard deviation of three measurements.

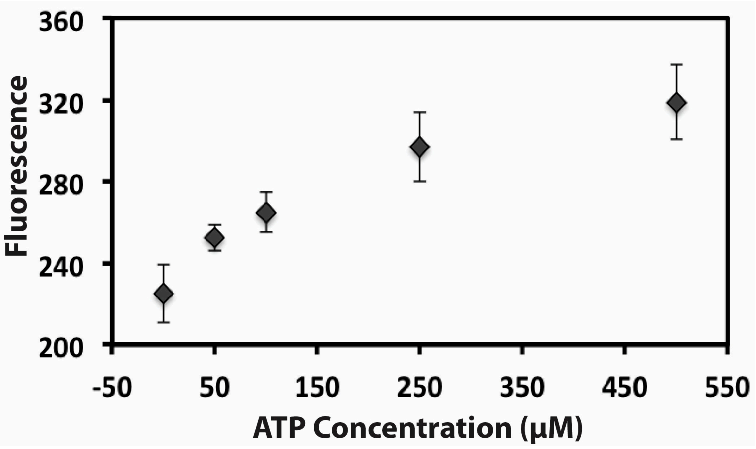

Figure S3. Fluorescence responses in real sample. The fluorescence responses for the biosensing system against ATP concentrations (0 µM, 50 µM, 100 µM, 250 µM, 500 µM respectively) in 10% human serum. The data are an average of three independent experiments.

© 2015 by the authors; licensee MDPI, Basel, Switzerland. This article is an open access article distributed under the terms and conditions of the Creative Commons Attribution license (http://creativecommons.org/licenses/by/4.0/).
